# Effect of mandibular advancement device on the stomatognathic system in patients with mild‐to‐moderate obstructive sleep apnoea‐hypopnoea syndrome

**DOI:** 10.1111/joor.12982

**Published:** 2020-06-11

**Authors:** Jing Zhou, De‐Hong Li, Peng‐Fei Zhu, Chun‐Yan Yi, Lin Chang, Yanan Zhang, Xiang‐Hong Yang

**Affiliations:** ^1^ Department of Stomatology Kunming Yan’an Hospital Kunming City China

**Keywords:** electromyography, MRI, mandibular advancement snore stopping device, mandibular locus, obstructive sleep apnoea‐hypopnoea syndrome, temporomandibular changes

## Abstract

**Objective:**

This study was conducted to evaluate the changes of temporomandibular joints (TMJs) through magnetic resonance imaging (MRI) scanning and the electrical changes in mandibular movement and masticatory muscle surface of mild‐to‐moderate obstructive sleep apnoea‐hypopnoea syndrome (OSAHS) patients before and after treatment with mandibular advancement device (MAD).

**Methods:**

This was a single‐centre, prospective study recruiting OSAHS patients undergoing treatment with MAD in Department of Stomatology, Yannan Hospital, Kunming, China. Patients were recruited from February 2015 to October 2015, and TMJ changes were observed in MRI scanning before and after 18 months of treatment with MAD in cohort 1. The second cohort of the patients were recruited from January 2014 to September 2015 and electrical changes in mandibular movement and masticatory muscle surface of patients before and after 6 months of treatment with MAD.

**Results:**

In the cohort 1, TMJ changes analysed through MRI scanning, before and after 18‐month treatment with MAD, there was no significant deviation in the angle of joint disc position. A minor change in the position relationship between condylar process, articular disc and articular fossa but not significant was observed. There was no significant difference in the shape and magnitude of mandibular incision edge movement, percussion movement, masticatory movement and condylar central trajectory among the recruited OSAHS patients, before and after 6 months of MAD treatment as analysed through electromyography.

**Conclusion:**

In this study, from the results it was evident that the effect of MAD on the stomatognathic system of OSAHS patients is minimal.

## 1 INTRODUCTION

Obstructive sleep apnoea–hypopnoea syndrome (OSAHS) is characterised by repetitive episodes of complete or partial upper airway obstruction during sleep which leads to snoring, intermittent hypoxaemia and sleep fragmentation.^1^ The global prevalence of obstructive sleep apnoea (OSA) has been reported to be approximately one billion in 2019, of which China has the highest number of individuals suffering from OSA, followed by Untied States, Brazil and India.^2^ This is an alarming situation for healthcare policymakers, since OSAHS is associated with frequent, excessive daytime sleepiness which may lead to increase in the number of vehicle crashes, occupational accidents and impaired quality of life (QOL).^1^

Continuous positive airway pressure (CPAP) and oral appliances like mandibular advancement device (MAD) are the two most widely used treatment strategies for OSAHS symptoms, of which CPAP has been shown to be very effective in reducing sleepiness, road accidents, cardiovascular risk and mortality.^1^, ^3^, ^4^ However, patient compliance is a major drawback of this device, and as per reports, approximately 29%‐83% of OSAHS patients were not able to use CPAP constantly, leading to decrease in the efficacy.^5^, ^6^, ^7^, ^8^, ^9^ MAD is yet another effective treatment strategy followed mainly for patients with mild to moderate cases of OSAHS.^10^ Although CPAP therapy has shown superior effect in improving the symptoms of OSAHS specially for reducing apnoea–hypopnoea index (AHI), patient acceptability and compliance have been found to be better with MADs with comparable quality of life (QoL).^11^ The MAD devices dilate the upper airway during sleep by holding the mandible in a forward position, which aids in clearing the airway and consequently reducing snoring..^1^ A practice parameter update, for the treatment of snoring and obstructive sleep apnoea (OSA) with mandibular advancement devices (MADs), recommended using MADs as first‐line therapy for patients with mild‐to‐moderate OSAHS, who preferred MADs to CPAP, or were not responding to CPAP therapy.^12^

Number of short‐term and long‐term qualitative analysis and questionnaire‐based studies have shown MAD to be efficacious in terms of reduced AHI as estimated by polysomnography, reduced frequency of snoring, decreased daytime sleepiness and reduced Epworth sleepiness score (ESS) in OSAHS patients.^1^, ^13^, ^14^, ^15^, ^16^, ^17^, ^18^ However, there have been concerns over the long‐term side effects of the device. Some of the studies involving OSAHS patients have reported changes in temporomandibular joints (TMJs) and the oro‐facial function due to the protruded jaw position during sleep.^19^, ^20^, ^21^ Thus, a need arises to assess the changes in stomatognathic system after treatment with MAD at different time points, since these devices are meant for long‐term or even lifetime use. Analysing the TMJ and mandibular changes with the aid of MRI scanning and electromyography (EMG) would provide an insight of the long‐term changes or side effects with the use of MAD, based on which preventative measures could be taken. This study was conducted to evaluate the changes of TMJs through magnetic resonance imaging (MRI) scanning and the electrical changes in mandibular movement and masticatory muscle surface of mild‐to‐moderate OSAHS patients before and after treatment with mandibular advancement device.

## 2 METHODS

### 2.1 Study design and patient population

This was a single‐centre, prospective study recruiting OSAHS patients undergoing treatment with MAD in Department of Stomatology, Yannan Hospital, Kunming, China. There were two cohorts of patients. In the first cohort, the patients were recruited from February 2015 to October 2015; temporomandibular changes were observed in MRI scanning before and after 18‐month treatment, with MAD. The second cohort of patients were recruited from January 2014 to September 2015 and electrical changes in mandibular movement and masticatory muscle surface of patients before and after 6 months treatment, with MAD. The study was approved from institutional ethics committee, and an informed consent form was obtained before enrolling the patients.

Patients with mild‐to‐moderate OSAHS, over 20 years of age, regardless of gender, with no less than 10 single jaw teeth, stationary periodontal disease, no lost teeth above I degree, recommended for MAD and adhering to wear MAD every night for more than 5 hours, were included in the study. Patient's severity of OSAHS was determined by the apnoea/ hypopnoea index (AHI), which is evaluated by polysomnography (PSG) tests. Patients were classified as mild, moderate or severe based on AHI and were considered to have mild OSAHS when AHI was 5‐15 times/hr with lowest oxygen saturation of 85%‐90%; moderate OSAHS when AHI was 15‐30 time/hr with lowest oxygen saturation of 65%‐85%; and severe OSAHS if AHI > 30 times/hr with lowest oxygen saturation of <65%.^22^

Patients with history of craniomaxillofacial trauma and operation, active periodontal disease, temporomandibular disease (TMD), nocturnal bruxism and those who were mentally unfit or were not comfortable were excluded from the study.

**Figure 1:**
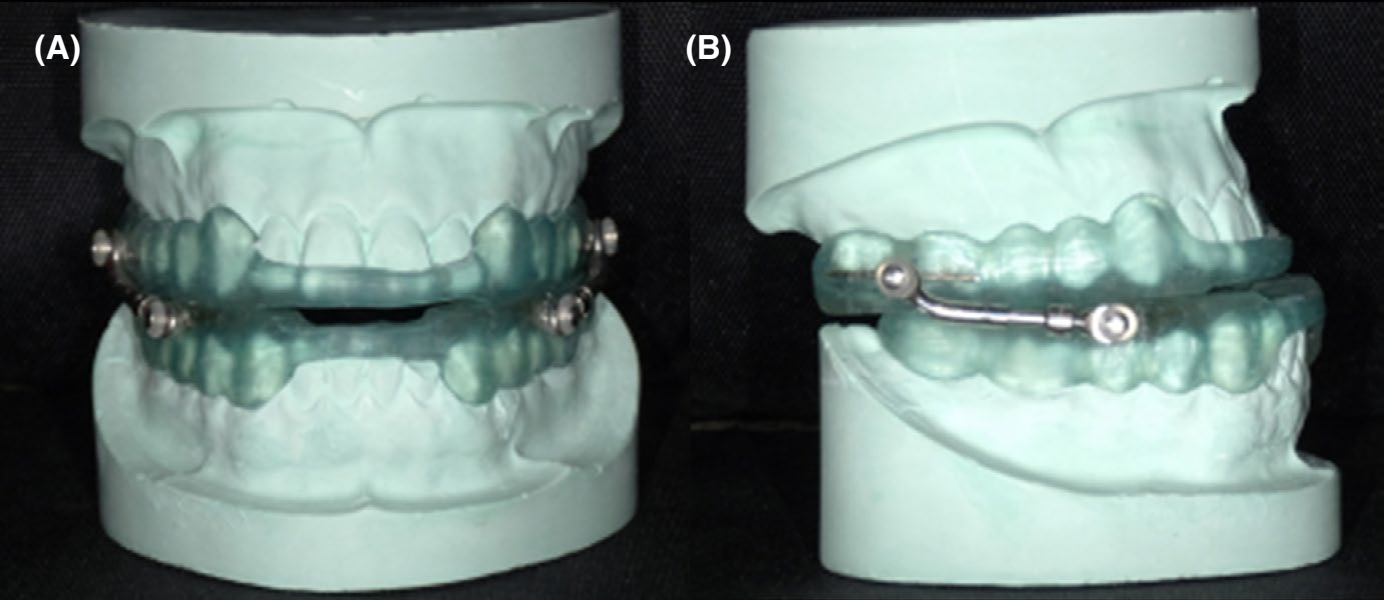
Mandibular protrusion snore arrester [Colour figure can be viewed at wileyonlinelibrary.com]

All the patients were treated with the split type MAD which could be positioned by adjusting a metal rod (Figure 1). The distance of mandibular protrusion in all patients was maintained at 75% of the maximum protrusion position, and the vertical distance of anterior teeth was 4‐5 mm. Patients were made asked to re‐visit after 1 day of wearing the MAD to determine the comfort level and were monitored till they were adapted with the device. Incremental retraction of 0.3 mm (0.3 mm/circle/time) was done until the patients felt comfortable at a personalised optimal forward extension position to improve compliance.

### 2.2 Outcomes analysed

For analysing the TMJ changes, we evaluated the structural changes in the articular disc, condyle and glenoid fossa. Further, the relative positions of articular disc and condyle from the glenoid fossa and articular disc from the condyle were analysed by MRI scanning before and after 18 months of MAD treatment in cohort 1. The soft tissue changes were evaluated in cohort 2 by electromyographic analysis of the masticator muscle by evaluating the different mandibular movement facilitated by the masticator muscle.

**Figure 2:**
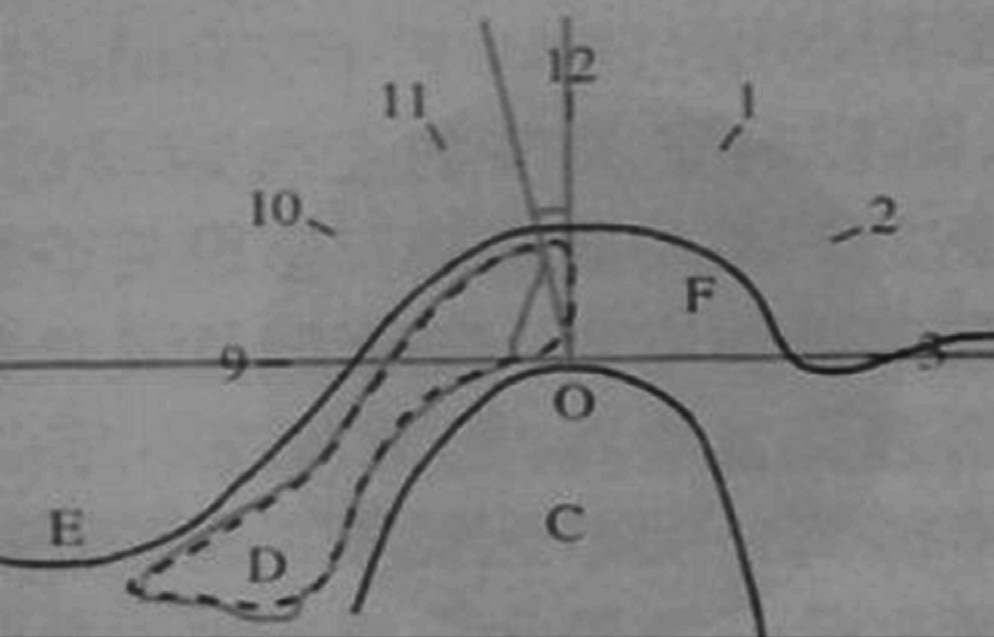
The clock method (Drace analysis) to evaluate joint disc position

**Figure 3:**
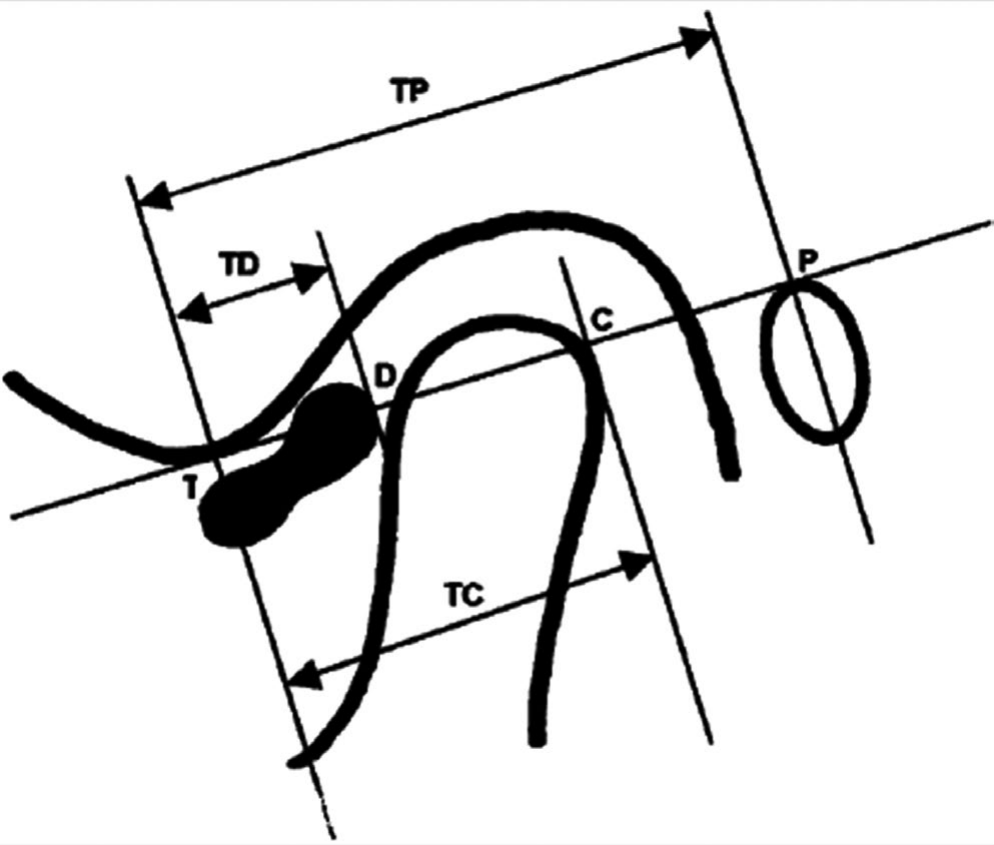
Schematic diagram of Kurita analysis method

Temporomandibular joint structure was measured at oblique sagittal and oblique coronal planes. In order to eliminate the internal errors of researchers and to improve the reliability of the research, all measurements were completed by the same researcher, and each index was measured three times, and its average value was taken as the measurement data. Surface flexible coil was selected and the patient was made to lie in a supine position on SIEMENS Verio 3.0T superconducting magnetic resonance instrument. The head sagittal plane was consistent with the long axis of the bed surface, and the earplug was put in place. The patient was asked to close his teeth, and the centre of the coil was aligned with the temporal head in front of the external auditory canal. All the patients underwent bilateral oblique sagittal and oblique coronal scans in closed position. Scanning of the transverse axis was used to locate the condyle. Oblique sagittal and oblique coronal planes were located at the level of the transverse axis showing the condyle. Oblique sagittal plane was located perpendicular to the long axis of the condyle (internal and external diameter of the condyle, Figure 2), and the scanning was performed with the following imaging parameters: T1‐weighted image (T1WI) TR: 476ms; TE: 11 ms; matrix: 20 × 256; slice thickness: 2.0 mm; spacing: 0.2 mm; FOV: 150 × 150; T2‐weighted image (T2WI) TR: 4200 ms;TE: 78 ms; matrix: 320 × 224; slice thickness: 2.0 mm; spacing: 0.2 mm; FOV: 150 × 150 and proton density‐weighted image (PDWI) TR: 3200 ms; TE: 32 ms; matrix: 320 × 224; slice thickness: 2.0 mm; spacing: 0.2 mm; FOV: 150 × 150. The oblique coronal plane was parallel to the long axis of the internal and external diameter of the temporomandibular condyle (Figure 3), and the scanning was performed with the following imaging parameters: T1‐weighted image (T1WI) TR: 4200 ms; TE: 78 ms; matrix: 320 × 256; slice thickness: 2.0 mm; spacing: 0.2 mm; FOV: 150 × 150; T2‐weighted image (T2WI) TR: 400 ms;TE: 11 ms; matrix: 320 × 224; slice thickness: 2.0 mm; spacing: 0.2 mm; FOV: 150 × 150 and proton density‐weighted image (PDWI) TR: 2800ms; TE: 33 ms; matrix: 320 × 224; slice thickness: 2.0 mm; spacing: 0.2 mm; FOV: 150 × 150. The parameters used for transverse axis scanning were as follows: repetition time (TR): 3570 ms; echo time (TE): 78ms; matrix: 256 × 256; slice thickness: 3.0 mm; spacing: 0.2 mm; scanning range (FOV): 179 × 179.

### 2.3 Evaluation of joint disc position

The clock method (Drace method) as shown in Figure 2 was used to evaluate the joint disc position. The analysis was performed as described earlier ^23^ with the closed oblique sagittal T1WI image which shows a clear demarcation line between the posterior disc zone and the bilateral plate zone (ie, the disc demarcation line). The preservation of the normal anatomical relationship after the long‐term usage of MAD is evaluated.

Kurita analysis, as described earlier,^24^ was used to study the positional changes of condylar process, articular disc and articular fossa with respect to each other before and after treatment as shown in Figure 3.

### 2.4 Oblique coronal plane measurement method

The PDW1 image taken from the occlusal oblique coronal plane, displaying the condyle and its neck, was used for this analysis as described earlier.^25^ In brief, two vertical lines were drawn on the inner and outer edges of the condyle and the articular disc, inner edge line A, C and the outer edge line B, D respectively. The distance between lines A and C is the distance between the inner edges of the disc and condyle, and the distance between lines B and D is the distance between the outer edges of the disc and condyle. The medial margin, line A, is negatively spaced at the inner edge of line C, while the lateral margin, line B, is negatively spaced at the outer edge of line D. If the distance between the inner edge of the disc and condyle is positive, it means that the inner edge of the disc exceeds the inner edge of the condyle inward and the outer edge of the disc represents that the outer edge of the disc exceeds the outer edge of the condyle outward.

### 2.5 Other observed indicators

The T1WI image from the oblique sagittal plane was analysed for the changes of condylar bone including condylar plane, osteophyte, cortical resorption and destruction, and the short condyle. With the T2WI image, high signal areas in the articular cavity denoting joint effusion were analysed.

### 2.6 Methods for evaluation of mandibular changes

#### 2.6.1 Instrument preparation

In this experiment, zinc phosphate was used to adhere the prepared magnetic steel to the middle one‐third surface of the labial side of the mandibular teeth; the proximal mid‐incisor from the mandibular central incisor was used as the experimental marker point, and the centre of the magnetic steel handle was positively positioned to the marker point; the magnetic steel was not always in contact with the maxillary dentition during the median occlusion, protrusion occlusion and lateral occlusion. The surface electromyogram was attached to the anterior bundle of temporal muscle and masseter muscle on both sides of the patient, respectively, and the facial arch was correctly placed in the external auditory canal and fixed on the head connected the sensor.

#### 2.6.2 Detection of mandibular motion trajectory

A mandibular trajectory tracer was used to detect the marginal trajectory of mandibular tangential points, the trajectory of kowtow, the trajectory of chewing and the motion amplitude of each axis, the trajectory of bilateral condyles, the inclination of forward and lateral non‐working condyles, and the Bennett angle of patients with OSAHS before and 6 months after MAD treatment. The mandibular movement modes recorded were as follows: mandibular incision margin movement (including maximum mouth opening movement; maximum forward movement; left and right lateral movement); percussion movement; mandibular chewing movement (chewing gum, left and right sides chewing for 30 seconds, respectively).

#### 2.6.3 Electromyography of masticatory muscles

Sterile cotton ball was dipped in 95% medical ethanol and used to degrease the skin at the test site. After the skin was dry, surface electromyograms were attached to the test site, surface electrodes were placed, and grounding wires were placed behind the neck. Simultaneously with mandibular motion trajectory detection, EMG signals of bilateral anterior temporal muscle bundle and masseter muscle surface were collected and recorded. For, bilateral anterior temporal muscle bundles the electrodes were placed at the outer auditory meatus edge forward about 6 cm, vertical upward about 6 cm, located on the orbital‐auricular plane. For, masseter muscle the electrodes were placed at external auditory meatus edge forward about 2.5 cm, vertical down about 6 cm, in the upper and anterior position of the mandibular angle.

### 2.7 Statistical analysis

All experimental data were processed by SPSS21.0 version. Paired *t* test and descriptive statistics were used in the analysis. The difference in mean values and percentages before and after treatment were analysed. *P* value of < 0.05 was considered to be statistically significant.

## 3 RESULTS

A total of twenty patients treated with MAD (Table 1) were included in each cohort (cohort 1 and 2). The participants were recruited between February 2015 and October 2015 (for Cohort 1) and January 2014 and September 2015 (for Cohort 2), in the Department of Stomatology, Yannan Hospital, Kunming, China. There were no dropouts due to non‐compliance with MAD.

**Table 1:** Socio‐demographic and clinical characteristics of the participants (mean ± SD, mm)

Parameters	Cohort 1	Cohort 2
Mean (SD) age	41.75 ± 6.23	44.73 ± 8.95
Gender		
Male:Female	7:3	8:2
APNOEA severity		
Mild	9	9
Moderate	11	11
Body mass index^a^	21.72 + 3.46	
Minimum SaO_2_%^a^	82.80 + 5.77	
AHI/ times. H‐1^a^	17.00 + 5.98	

^a^ Provided for the combined set of patients.

### 3.1 Drace analysis results

**Figure 4:**
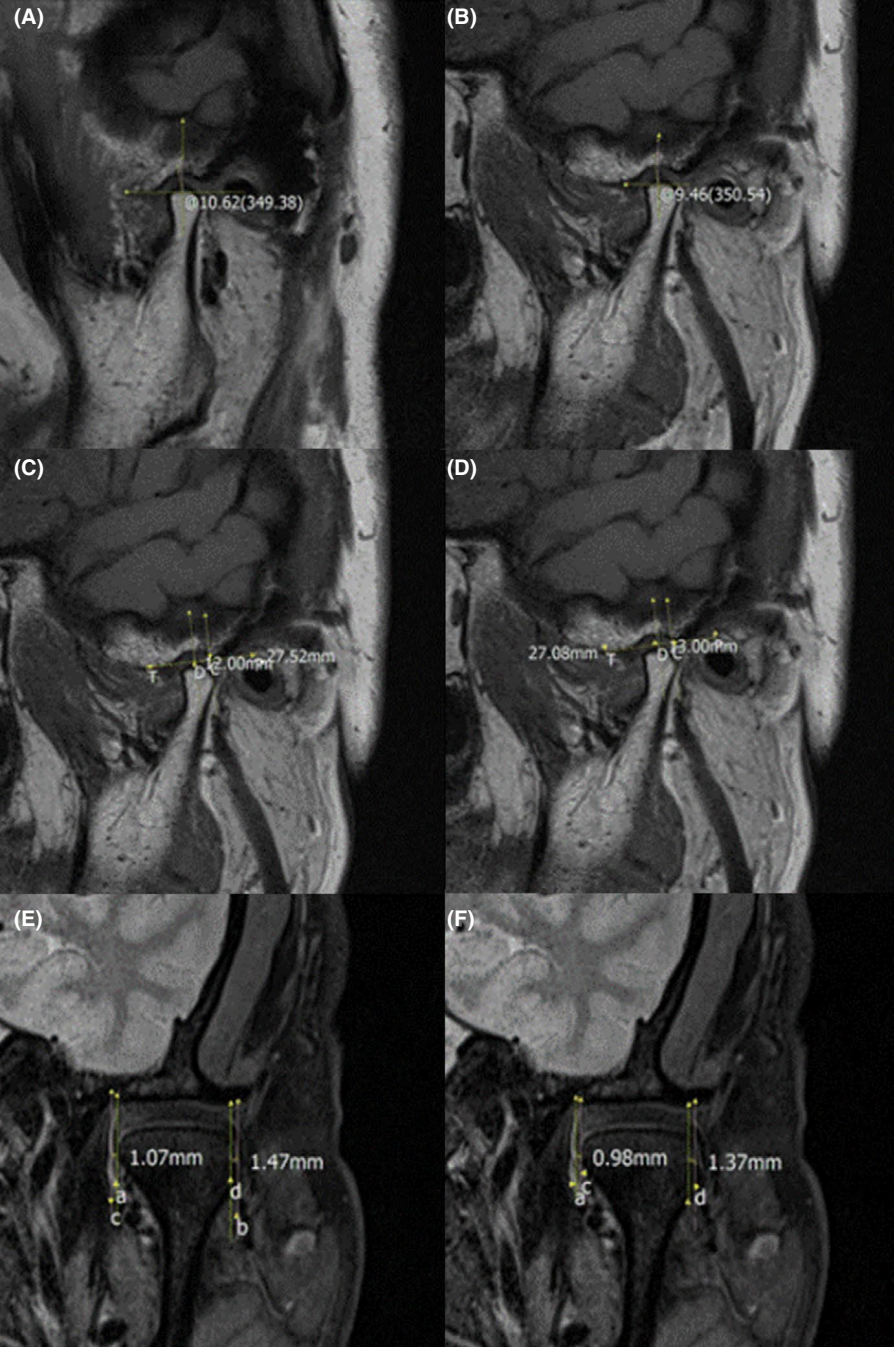
Temporomandibular changes before and after treatment with MAD. A and B, MRI obtained with Drace analysis. C and D, MRI obtained with Kurita analysis. E and F, Measurements of oblique coronal position [Colour figure can be viewed at wileyonlinelibrary.com]

After 18 months of MAD treatment, there was no significant deviation in the degree of the angle. Table 2; Figure 4A,B.

**Table 2:** Measurement results from MRI images

Parameters	Before treatment (Mean ± SD)	After treatment (Mean ± SD)	*T* values	*P* values
Drace analysis—location of articular disc before and after treatment				
Left side (**°)**	8.50 ± 2.07	8.92 ± 1.50	1.38	0.19
Right side (**°)**	8.53 ± 1.15	8.95 ± 1.39	1.65	0.12
Kurita analysis measurement				
TD/TP	0.41 + 0.06	0.40 + 0.06	0.35	0.74
TC/TP	0.58 + 0.05	0.56 + 0.06	0.85	0.42
TD/TC	0.70 + 0.07	0.70 + 0.08	0.19	0.85
Oblique coronal measurements				
Inner edge spacing	0.54 ± 1.37	0.63 ± 1.41	0.81	0.42
Outer edge spacing	−1.55 ± 1.16	−1.73 ± 1.23	1.42	0.17

Note

No significant difference between before and after treatment, *P* > .05.

### 3.2 Measurement results by Kurita method

The analysis of positional relationship between condylar process, articular disc and articular fossa revealed non‐significant difference in the position of condylar process and articular disc with reference to articular fossa. Similarly, there was no significant difference in the position of articular disc and condylar process before and after treatment (Table 2; Figure 4C,D).

### 3.3 Measurements of oblique coronal position

For the coronal measurement of oblique closure, the inner edge of the articular disc went inward beyond the inner edge of the condyle and the outer edge of the disc did not exceed the outer edge of the condyle. There was no significant difference between the two groups before and after treatment for both inner and outer edge spacing (*P* > .05). (Table 2; Figure 4E,F).

After 6 months of MAD treatment, the movement track of mandibular incisor opening and closing had high consistency, good repeatability, no significant difference before and after treatment, no left‐right deviation on coronal plane and horizontal plane; the maximum movement track of mandibular incisor opening was mainly smooth curve in coronal plane, sagittal plane and horizontal plane, and the smoothness was mainly smooth curve in treatment. There was no significant difference before and after treatment, and there was no significant difference between the open and closed types of subjects before and after treatment. Before and after treatment, the opening trajectory on sagittal plane was located behind the closing trajectory, and the opening trajectory and closing trajectory were separated in the front and middle part of the trajectory, and the degree of separation was comparatively consistent before and after treatment. The mandibular tangential point forward movement had good repeatability and smoothness in the horizontal and sagittal trajectory curves, and there was no significant difference between the left and right margins of the mandibular tangential point before and after treatment. The symmetry and smoothness of the curve on the coronal plane are good. The derailment curve and the entry curve are partially separated, but the symmetry, smoothness and separation degree of the curve are consistent before and after treatment.

### 3.4 Quantitative analysis of three‐dimensional trajectory of mandibular tangential edge motion

#### 3.4.1 Maximum mouth opening track amplitude at mandibular incision point

After 6 months of MAD treatment, the maximum trajectory amplitude of mandibular incisal opening did not change in the horizontal direction of coronal plane, but decreased slightly in the vertical direction of sagittal plane. There was no significant difference in the trajectory amplitude of mandibular incisal maximal mouth opening on horizontal, sagittal and coronal planes before and after treatment for 6 months (*P* > .05). Table 3.

**Table 3:** Quantitative analysis of three‐dimensional trajectory of mandibular tangential edge motion

Parameters	Coronal plain (mm)	Sagittal plain (mm)	Horizontal plain (mm)
Maximum opening trajectory amplitude at mandibular tangent point			
Before treatment (mean ± SD)	31.07 ± 2.58	23.60 ± 7.21	1.91 ± 0.61
After treatment (mean ± SD)	31.10 ± 2.57	23.64 ± 7.10	1.88 ± 0.61
*T* values	1.823	0.450	1.584
*P* values	0.084	0.658	0.130
The range of forward motion trajectory at mandibular tangential point			
Before treatment (mean ± SD)	3.92 ± 0.85	8.14 ± 1.93	1.47 ± 0.43
After treatment(mean ± SD)	3.93 ± 0.83	7.68 ± 2.29	1.47 ± 0.44
*T* values	0.937	1.026	0.380
*P* values	0.361	0.318	0.708
Track amplitude of left margin of mandibular tangential point			
Before treatment (mean ± SD)	3.99 ± 0.49	2.46 ± 0.85	6.92 ± 1.21
After treatment (mean ± SD)	3.98 ± 0.51	2.47 ± 0.83	6.94 ± 1.20
*T* values	0.016	0.857	2.001
*P* values	0.987	0.402	0.060
The range of the right margin motion trajectory of the mandibular tangential point			
Before treatment (mean ± SD)	3.83 ± 0.72	2.07 ± 0.64	6.80 ± 1.12
After treatment (mean ± SD)	3.82 ± 0.71	2.08 ± 0.64	6.81 ± 1.10
*T* values	0.371	1.791	0.849
*P* values	0.71	0.089	0.406

Note

There was no significant difference before and after treatment, *P* > .05.

#### 3.4.2 Track amplitude of mandibular tangential protrusion

After 6 months of MAD treatment, the trajectory amplitude of mandibular incision protrusion did not change horizontally, but slightly decreased horizontally in sagittal plane. There was no significant difference in the trajectory amplitude of mandibular incision protrusion on horizontal, sagittal and coronal planes before and after treatment for 6 months (*P* > .05). Table 3.

#### 3.4.3 Contrast amplitude of mandibular tangential protrusion locus

After 6 months of MAD treatment, the left margin of mandibular incision increased slightly in the horizontal direction of the coronal plane and decreased slightly in the vertical direction of the horizontal plane. Before treatment and 6 months after treatment, there was no significant difference in the trajectory amplitude of the left margin movement of mandibular incision on horizontal, sagittal and coronal planes (*P* > .05). Table 3.

#### 3.4.4 Track amplitude of the right margin of mandibular tangential point

Six months after MAD treatment, the right margin of mandibular incision increased slightly horizontally in the coronal plane and decreased slightly vertically in the horizontal plane. There was no significant difference in the trajectory amplitude of the right margin movement of mandibular incision in horizontal, sagittal and coronal planes before and after treatment for 6 months (*P* > .05). Table 3.

### 3.5 Quantitative analysis of three‐dimensional motion amplitude of mandibular incision chewing

#### 3.5.1 Three‐dimensional amplitude of left chewing movement at mandibular incision

After 6 months of MAD treatment, the amplitude of left chewing locus at mandibular incision increased slightly in coronal plane, and the amplitude at sagittal plane was almost the same. There was no significant difference in horizontal, sagittal, coronal and coronal masticatory ring width between subjects before MAD and 6 months after MAD treatment (*P* > .05), Table 4.

**Table 4:** Quantitative analysis of three‐dimensional motion amplitude of mandibular incision chewing

Parameters	Coronal plane (mm)	Sagittal plane (mm)	Horizontal plane (mm)	Coronal width(mm)
The range of left chewing locus at mandibular cut point				
Before treatment (mean ± SD)	13.58 ± 3.64	6.02 ± 2.20	3.13 ± 1.56	4.09 ± 1.20
After treatment (mean ± SD)	13.59 ± 3.61	6.07 ± 2.26	3.13 ± 1.55	4.14 ± 1.24
*T* values	1.764	0.809	0.230	1.157
*P* values	0.09	0.428	0.821	0.262
The range of right chewing locus at mandibular cut point				
Before treatment (mean ± SD)	13.58 ± 3.64	6.02 ± 2.20	3.58 ± 1.95	4.09 ± 1.35
After treatment (mean ± SD)	13.59 ± 3.61	6.07 ± 2.26	3.58 ± 1.93	4.15 ± 1.39
*T* values	1.764	0.809	0.580	1.127
*P* values	0.094	0.428	0.569	0.274

Note

There was no significant difference before and after treatment, *P* > .05.

#### 3.5.2 Three‐dimensional amplitude of right chewing movement at mandibular incision

After 6 months of MAD treatment, the range of left chewing locus at mandibular incision increased slightly in coronal plane, sagittal plane and horizontal plane. There was no significant difference in horizontal, sagittal, coronal and coronal masticatory ring width between the right side of mandibular incision before and 6 months after MAD treatment (*P* > .05), Table 4.

### 3.6 Mandibular margin motion, central motion of condyle and lateral condylar inclination of protrusion and sagittal plane

#### 3.6.1 Movements of mandibular margin and central motion of condyle

When the mandibular condylar process centre was in the maximum opening movement, the repeatability of derailment and entry curve was good, the separation degree of sagittal alignment curve was consistent before and after treatment, the movement direction was stable, the trajectory was smooth on the coronal, horizontal and sagittal planes, and there was no significant difference before and after treatment. The motion amplitude of the working side was smaller than that of the non‐working side, while the sagittal plane was smooth. The upper short line part represents the central motion trajectory of the lateral condyle of the working side during the left and right lateral movement. The trajectory figure of the working side is small and unclear, while the trajectory of the lateral condyle of the non‐working side is clear, stable and reproducible. The trajectory amplitude of the lateral motion and the forward motion is obviously smaller than the maximum open motion amplitude. The central trajectory of bilateral condyles was symmetrical; the curve was smooth and reproducible. The trajectory shape, smoothness and reproducibility of bilateral condyles were consistent before treatment and 6 months after MAD treatment.

### 3.7 Bilateral anterior extension condylar inclination, non‐working lateral condylar inclination (sagittal plane) and Bennett angle measurement (horizontal plane)

#### 3.7.1 Left protrusion, non‐working lateral condylar slope and Bennett angle

There was no significant difference in left anterior extension, lateral condylar slope and Bennett angle between the mild and moderate OSAHS patients before and 6 months after MAD treatment (*P* > .05), Table 5.

**Table 5:** Bilateral anterior extension condylar inclination, non‐working lateral condylar inclination (sagittal plane) and Bennett angle measurement (horizontal plane)

Parameters	Prolonged condylar inclination (°)	Sagittal non‐working side condylar inclination (°)	Bennet angle (°)
Left protrusion, non‐working lateral condylar slope and Bennett angle			
Before Treatment (mean ± SD)	36.22 ± 0.57	41.02 ± 1.24	4.40 ± 0.37
After Treatment (mean ± SD)	36.23 ± 0.56	41.02 ± 1.23	4.40±0.36
*T* values	1.235	1.584	0.623
*P* values	0.232	0.130	0.541
Right protrusion, sagittal non‐working lateral condylar slope and Bennett angle			
Before treatment (mean ± SD)	36.22 ± 0.57	41.02 ± 1.24	4.40 ± 0.37
After treatment (mean ± SD)	36.23 ± 0.56	41.02 ± 1.23	4.40±0.36
*T* values	1.235	1.584	0.623
*P* values	0.232	0.130	0.541

Note

There was no significant difference before and after treatment, *P* > .05.

#### 3.7.2 Right protrusion, non‐working lateral condylar slope and Bennett angle

There was no significant difference in right protrusion, lateral condylar inclination and Bennett angle between patients with mild and moderate OSAHS before and 6 months after MAD treatment (*P* > .05), Table 5.

Six months after MAD treatment, the forward motion track of mandibular tangential point decreased slightly in the centre of left condyle on sagittal plane, and the left condyle inclination decreased slightly. The symmetry of left and right condyle was comparable before and after treatment.

Six months after MAD treatment, the inclination of the non‐working lateral condyle in the sagittal trajectory decreased slightly, while that in the sagittal trajectory increased slightly when the mandibular tangential point moved to the right. The symmetry of the non‐working lateral condyle in the sagittal trajectory was comparable before and after treatment.

After 6 months of MAD treatment, the Bennett angle in the horizontal trajectory decreased slightly while the left and right side of mandibular incision moved, and the Bennett angle was positive.

### 3.8 Analysis of other indicators of TMJ changes observed by MRI scanning

The analysis of TMJ changes after 18 months of MAD usage revealed no hyperplasia of condylar bone and smooth surface and no change of condylar bone when observed in oblique sagittal T1WI MRI scanning. Also, no joint effusion was found in any of the patients enrolled when observed with T2WI MRI scanning.

### 3.9 Changes of surface electromyography of temporalis anterior bundle and masseter muscle of mandibular movement

#### 3.9.1 Qualitative description of the changes of EMG of mandibular margin movement, masticatory movement, anterior temporal muscle bundle and masseter muscle surface before and after treatment

Before and 6 months after MAD treatment, the regularity of EMG cycle in mandibular marginal movement and masticatory movement was consistent, the coordination between unilateral masseter and temporal muscle was consistent, the symmetry of bilateral homonyms was consistent, and the alternation of EMG peaks and troughs were stable and consistent.

### 3.10 Comparisons of electromyography peak values of mandibular marginal movement, masticatory movement, anterior temporal muscle bundle and masseter muscle surface before and after treatment

#### 3.10.1 Comparison of peak electromyogram of masticatory muscles during mandibular maximum mouth opening exercise

There was no significant difference in the peak value of masticatory myoelectricity in patients with mild and moderate OSAHS before and 6 months after MAD treatment (*P* > .05). (Table 6).

**Table 6:** Comparisons of electromyography peak values for mandibular marginal movement, masticatory movement, anterior temporal muscle bundle and masseter muscle surface

Parameters	LMV (mv)	RMM (mv)	LTA (mv)	RTA (mv)
Peak electromyogram of masticatory muscles during maximum mouth opening exercise at mandibular cut point				
Before treatment (mean ± SD)	59.45 ± 11.03	74.00 ± 12.57	60.00 ± 12.54	58.70 ± 13.81
After treatment (mean ± SD)	59.30 ± 10.98	73.80 ± 12.77	59.80 ± 12.44	57.60 ± 12.93
*T* values	0.900	1.453	1.073	1.287
*P* values	0.379	0.163	0.297	0.214
Peak electromyogram of masticatory muscles during mandibular protrusion exercise				
Before treatment (mean ± SD)	50.10 ± 12.99	55.60 ± 15.59	57.85 ± 13.68	56.85 ± 10.25
After treatment (mean ± SD)	50.00 ± 13.06	55.40 ± 15.78	57.70 ± 13.76	56.60 ± 10.24
*T* values	0.623	1.453	1.831	1.751
*P* values	0.541	0.163	0.083	0.096
Peak electromyogram of masticatory muscles during left lateral mandibular movement				
Before treatment (mean ± SD)	75.90 ± 13.09	50.95 ± 10.12	72.05 ± 13.24	52.15 ± 5.82
After treatment (mean ± SD)	75.75 ± 13.04	50.75 ± 10.19	71.75 ± 13.24	52.00 ± 5.84
*T* values	1.831	1.710	2.042	1.000
*P* values	0.083	0.104	0.055	0.33005
Peak electromyogram of masticatory muscles during right lateral mandibular movement				
Before treatment (mean ± SD)	31.55 ± 6.62	80.30 ± 12.08	50.75 ± 8.81	78.70 ± 9.57
After treatment (mean ± SD)	31.60 ± 6.77	80.15 ± 12.17	50.65 ± 8.77	78.65 ± 9.58
*T* values	0.370	1.831	0.698	0.370
*P* values	0.716	0.083	0.494	0.716
Peak electromyogram of masticatory muscles during left mandibular chewing exercise				
Before treatment (mean ± SD)	192.75 ± 13.87	94.20 ± 7.74	169.85 ± 14.00	69.10 ± 11.46
After treatment (mean ± SD)	192.80 ± 13.79	94.25 ± 7.57	169.60 ± 14.02	69.20 ± 11.47
*T* values	‐0.370	‐0.295	1.751	‐0.698
*P* values	0.716	0.772	0.096	0.494
Peak electromyogram of masticatory muscles during right mandibular chewing exercise				
Before treatment (mean ± SD)	84.60 ± 10.19	194.85 ± 11.13	67.60 ± 10.90	178.5 ± 12.36
After treatment (mean ± SD)	84.70 ± 10.18	194.90 ± 11.28	67.70 ± 10.54	178.65 ± 12.37
*T* values	0.809	0.370	0.698	1.831
*P* values	0.428	0.716	0.494	0.083

Note

There was no significant difference before and after treatment, *P* > .05.

LMM, left masseter; RMM, right masseter; LTA, left anterior temporalis; RTA, right anterior temporalis

#### 3.10.2 Comparison of peak electromyogram of masticatory muscles during mandibular protrusion exercise

There was no significant difference in the peak value of masticatory myoelectricity between patients with mild and moderate OSAHS before and 6 months after MAD treatment (*P* > .05). (Table 6).

#### 3.10.3 Comparison of peak electromyogram of masticatory muscles during left lateral mandibular movement

There was no significant difference in the peak value of masticatory myoelectricity between patients with mild and moderate OSAHS before and 6 months after MAD treatment (*P* > .05). (Table 6).

#### 3.10.4 Comparison of peak electromyogram of masticatory muscles during right lateral mandibular movement

There was no significant difference in the peak value of masticatory myoelectricity between patients with mild and moderate OSAHS before and 6 months after MAD treatment (*P* > .05). (Table 6).

#### 3.10.5 Comparison of peak electromyogram of masticatory muscles during left mandibular chewing exercise

There was no significant difference in the peak value of masticatory myoelectricity between patients with mild and moderate OSAHS before and 6 months after MAD treatment (*P* > .05). (Table 6).

#### 3.10.6 Comparison of peak electromyogram of masticatory muscles during right mandibular chewing exercise

For right mandibular mastication before and 6 months after MAD treatment in a patient with mild or moderate OSAHS, there was no significant difference in peak EMG of masticatory muscles during exercise (*P* > .05). (Table 6).

## 4 DISCUSSION

We present the anatomical changes in the form of TMJs and mandibular changes observed after the use of MAD by mild‐to‐moderate OSAHS patients in this study. For the TMJ changes analysed through MRI scanning, after 18 months of treatment with MAD, there was no significant deviation in the angle of joint disc position. A minor change which was not statistically significant was observed in the positional relationship between condylar process, articular disc and articular fossa. In addition to this, no significant changes in inner and outer edge spacing were observed before and after treatment with MAD.

There are several reports for temporomandibular side effects like jaw or facial muscle pain, changes in occlusion like change in incisor inclination and decrease in overjet and overbite, TMJ pain, related with the use of MADs.^26^, ^27^, ^28^, ^29^, ^30^ However, there are studies which reported no significant side effects with long‐term use of these devices.^19^, ^20^, ^31^, ^32^, ^33^ A study assessed the MADs for the treatment of snoring and OSA for more than five years and reported that 95% were satisfied with the treatment and reported a non‐significant change in the TMJs after the use of the device.^33^ A questionnaire‐based long‐term study, evaluating the symptoms of temporomandibular dysfunction (TMD) in patients with OSA treated with MADs showed a significantly decreased TMD symptoms throughout treatment (*P* < .01).^32^ A cephalometric analysis of cranio‐facial changes for the long‐term use of MAD for OSA revealed no significant changes in skeletal variables.^20^ In line with the above reports, our study also reports non‐significant TMJ changes with long‐term use of MAD. Thus, it may be inferred that MAD may be safe for long‐term use in OSAHS.

A recent systematic review and meta‐analysis evaluating the effects of MAD involving OSA patients both with and without TMD signs and symptoms at baseline reveal that patients with prior signs and symptoms of TMD do not experience a significant increase in the symptoms due to MAD use and also suggest that presence of TMD need not be a routine contraindication for the use of MADs in OSA patient's management. However, in our study, patients with the history of TMD were not included.^34^ Another study assessing the long‐term dental and skeletal side effects of MAD therapy suggest that though the dental side effects small, they are clinically relevant and patients should be informed prior and monitored regularly.^35^

There are many studies reporting the side effects of MADs on the TMJs, but to the best of our knowledge, there is scarcity of reports for long‐term TMJ changes examined through MRI. A study assessed the changes in TMJs morphology and condyle position after the insertion of MAD for 11.5 months, with the aid of MRI scanning. No observable remodelling, morphology change and signal intensity change in the TMJs were noted in the MRI during the study period.^36^ However, there are few studies which indicate that there may be some degree of TMJs and condylar changes, remodelling of the articular tuberosity and disc displacement due to the long‐term use of MADs observed in MRI.^37^, ^38^ In our study, the MAD showed no significant change in the TMJs, but long‐term studies for two or more years with a greater sample size are still required to determine whether there will be any remodelling of the TMJs or neighbouring structures. In addition to this, there are reports for alterations in the masticatory muscles associated with the use of MADs.^28^, ^29^

The activity of masticatory muscles can be analysed with the help of EMG both in the subjects with normal occlusion and altered occlusion.^39^ On this principle, in this study, EMG was used to record the tapping movement, marginal movement, masticatory movement, condylar centre movement and surface electromyography of masticatory muscles in patients with mild‐to‐moderate OSAHS before and after MAD treatment for 6 months. To the best of our knowledge, it is the first report analysing the above parameters with the aid of EMG in the OSAHS patients using MAD. There was no significant difference in the shape and magnitude of mandibular incision edge movement, percussion movement, masticatory movement and condylar central trajectory among the recruited OSAHS patients, before and after 6 months of MAD treatment. In addition to this, the shape of masticatory ring at mandibular incision point, regularity and coordination of EMG of masticatory muscles after 6 months treatment was consistent with those before treatment. A study determining the pressure pain thresholds (PPTs) of masticatory and neck muscles change after the application of MAD in patients with OSA showed no significant difference in the physiological functions of the masticatory muscle and neck muscles after 6 months of MAD treatment.^40^ Similar to our study findings, another study also reported no greater changes in mandibular movements compared to control group after wearing MAD.^41^ However, in our study changes in mandibular movement were evaluated using EMG.

There are some limitations to this study. First, the sample size was too small. Second, follow‐up time for both TMJ changes (18 months), mandibular locus and masticatory muscle changes (6 months) was relatively shorter. Thirdly, although MRI is considered as a comprehensive technique to evaluate TMJ (disc position, effusion, gross condylar changes, etc), considering the short follow‐up period and minor alteration in bone structures and form, more sensitive technique like CT/CBCT could provide subtle signs of degenerative joint disease.^42^ Additionally, due to polysomnography expenses and lack of patient compliance, we completed the analysis of only 20 patients in each cohort of the study. The TMJ changes in patients with predisposing conditions were not evaluated in the study. Also, the TMJ changes and EMG changes were not evaluated in the same cohort. Owing to the limited data for side effects for MAD in OSAHS patients from MRI scanning and electromyography, this study may provide preliminary data set for future research.

## 5 CONCLUSION

From our study, it was evident that the effect of MAD on the stomatognathic system is minimal. However, since our study evaluated the outcomes for a shorter duration, further assessment evaluating the impact on a longer duration and on large cohort is warranted.

## CONFLICT OF INTEREST

The authors declare that there are no conflicts of interest.

## References

[1] Vecchierini M‐F , Attali V , Collet J‐M , et al. A custom‐made mandibular repositioning device for obstructive sleep apnoea‐hypopnoea syndrome: the ORCADES study. Sleep Med. 2016;19:131–140. 26364869 10.1016/j.sleep.2015.05.020

[2] Benjafield AV , Ayas NT , Eastwood PR , et al. Estimation of the global prevalence and burden of obstructive sleep apnoea: a literature‐based analysis. Lancet Res Med. 2019;7(8):687–698. 10.1016/S2213-2600(19)30198-5 PMC7007763 31300334

[3] Antic NA , Catcheside P , Buchan C , et al. The effect of CPAP in normalizing daytime sleepiness, quality of life, and neurocognitive function in patients with moderate to severe OSA. Sleep. 2011;34:111–119. 21203366 10.1093/sleep/34.1.111 PMC3001789

[4] Barbé F , Durán‐Cantolla J , Sánchez‐de‐la‐Torre M , et al. Effect of continuous positive airway pressure on the incidence of hypertension and cardiovascular events in nonsleepy patients with obstructive sleep apnea: a randomized controlled trial. JAMA [Internet]. 2012 [cited 2019 Jul 15];307. Available from: http://jama.jamanetwork.com/article.aspx?doi=10.1001/jama.2012.4366. 10.1001/jama.2012.4366 22618923

[5] Kribbs NB , Pack AI , Kline LR , et al. Objective measurement of patterns of nasal CPAP use by patients with obstructive sleep apnea. Am Rev Respir Dis. 1993;147:887–895. 8466125 10.1164/ajrccm/147.4.887

[6] Lindberg E , Berne C , Elmasry A , Hedner J , Janson C . CPAP treatment of a population‐based sample–what are the benefits and the treatment compliance? Sleep Med. 2006;7:553–560. 16740408 10.1016/j.sleep.2005.12.010

[7] Gagnadoux F , Le Vaillant M , et al. Influence of marital status and employment status on long‐term adherence with continuous positive airway pressure in sleep apnea patients. PLoS ONE. 2011;6:e22503. 21857929 10.1371/journal.pone.0022503 PMC3157341

[8] Weaver TE , Grunstein RR . Adherence to continuous positive airway pressure therapy: the challenge to effective treatment. Proc Am Thorac Soc. 2008;5:173–178. 18250209 10.1513/pats.200708-119MG PMC2645251

[9] Russo‐Magno P , O’Brien A , Panciera T , Rnp R , Rounds S . Compliance with CPAP therapy in older men with obstructive sleep apnea. J Am Geriatr Soc. 2001;49:1205–1211. 11559380 10.1046/j.1532-5415.2001.49238.x

[10] Marklund M , Stenlund H , Franklin KA . Mandibular advancement devices in 630 men and women with obstructive sleep apnea and snoring. Chest. 2004;125:1270–1278. 15078734 10.1378/chest.125.4.1270

[11] Almeida FR , Henrich N , Marra C , et al. Patient preferences and experiences of CPAP and oral appliances for the treatment of obstructive sleep apnea: a qualitative analysis. Sleep Breath. 2013;17:659–666. 22833346 10.1007/s11325-012-0739-6

[12] Kushida CA , Morgenthaler TI , Littner MR , et al. Practice parameters for the treatment of snoring and Obstructive Sleep Apnea with oral appliances: an update for 2005. Sleep. 2006;29:240–243. 16494092 10.1093/sleep/29.2.240

[13] Attali V , Chaumereuil C , Arnulf I , et al. Predictors of long‐term effectiveness to mandibular repositioning device treatment in obstructive sleep apnea patients after 1000 days. Sleep Med. 2016;27–28:107–114. 10.1016/j.sleep.2016.10.004 27938910

[14] Friedman M , Pulver T , Wilson MN , et al. Otolaryngology office‐based treatment of obstructive sleep apnea‐hypopnea syndrome with titratable and nontitratable thermoplastic mandibular advancement devices. Otolaryngol Head Neck Surg. 2010;143:78–84. 20620623 10.1016/j.otohns.2010.03.025

[15] Phillips CL , Grunstein RR , Darendeliler MA , et al. Health outcomes of continuous positive airway pressure versus oral appliance treatment for obstructive sleep apnea: a randomized controlled trial. Am J Respir Crit Care Med. 2013;187:879–887. 23413266 10.1164/rccm.201212-2223OC

[16] Gotsopoulos H , Kelly JJ , Cistulli PA . Oral appliance therapy reduces blood pressure in obstructive sleep apnea: a randomized, controlled trial. Sleep. 2004;27:934–941. 15453552 10.1093/sleep/27.5.934

[17] Doff MHJ , Hoekema A , Wijkstra PJ , et al. Oral appliance versus continuous positive airway pressure in obstructive sleep apnea syndrome: a 2‐year follow‐up. Sleep. 2013;36:1289–1296. 23997361 10.5665/sleep.2948 PMC3738037

[18] Brette C , Ramanantsoa H , Renouardiere J , Renouardiere R , Roisman G , Escourrou P . A mandibular advancement device for the treatment of obstructive sleep apnea: Long‐term use and tolerance. Int Orthodont. 2012;10:363–376. 10.1016/j.ortho.2012.09.001 23122735

[19] Martínez‐Gomis J , Willaert E , Nogues L , Pascual M , Somoza M , Monasterio C . Five years of sleep apnea treatment with a mandibular advancement device. Side effects and technical complications. Angle Orthod. 2010;80:30–36. 19852636 10.2319/030309-122.1 PMC8978723

[20] Doff MHJ , Hoekema A , Pruim GJ , Huddleston Slater JJR , Stegenga B . Long‐term oral‐appliance therapy in obstructive sleep apnea: a cephalometric study of craniofacial changes. J Dent. 2010;38:1010–1018. 20831889 10.1016/j.jdent.2010.08.018

[21] de Almeida FR , Lowe AA , Sung JO , Tsuiki S , Otsuka R . Long‐term sequellae of oral appliance therapy in obstructive sleep apnea patients: Part 1. Cephalometric analysis. Am J Orthod Dentofac Orthop. 2006;129:195–204. 10.1016/j.ajodo.2005.10.001 16473711

[22] Sleep‐related breathing disorders in adults: recommendations for syndrome definition and measurement techniques in clinical research. The Report of an American Academy of Sleep Medicine Task Force. 1999;22:667–689. 10450601

[23] Drace JE , Enzmann DR . Defining the normal temporomandibular joint: closed‐, partially open‐, and open‐mouth MR imaging of asymptomatic subjects. Radiology. 1990;177:67–71. 2399340 10.1148/radiology.177.1.2399340

[24] Kurita H , Ohtsuka A , Kobayashi H , Kurashina K . A study of the relationship between the position of the condylar head and displacement of the temporomandibular joint disk. Dentomaxillofac Radiol. 2001;30:162–165. 11420629 10.1038/sj/dmfr/4600603

[25] Schmitter M , Kress B , Ludwig C , Koob A , Gabbert O , Rammelsberg P . Temporomandibular joint disk position assessed at coronal MR imaging in asymptomatic volunteers. Radiology. 2005;236:559–564. 16040913 10.1148/radiol.2361040223

[26] Clark GT , Sohn J‐W , Hong CN . Treating obstructive sleep apnea and snoring: assessment of an anterior mandibular positioning device. J Am Dent Associat. 2000;131:765–771. 10.14219/jada.archive.2000.0275 10860328

[27] Tegelberg A , Wilhelmsson B , Walker‐Engström ML , et al. Effects and adverse events of a dental appliance for treatment of obstructive sleep apnoea. Swed Dent J. 1999;23:117–126. 10591454

[28] Pantin CC , Hillman DR , Tennant M . Dental side effects of an oral device to treat snoring and obstructive sleep apnea. Sleep. 1999;22:237–240. 10201069 10.1093/sleep/22.2.237

[29] Näpänkangas R , Raunio A , Sipilä K , Raustia A . Effect of mandibular advancement device therapy on the signs and symptoms of temporomandibular disorders. JOMR [Internet]. 2012 [cited 2019 Jul 23];3. Available from: http://www.ejomr.org/JOMR/archives/2012/4/e5/v3n4e5ht.htm. 10.5037/jomr.2012.3405 PMC3886094 24422023

[30] Doff MHJ , Veldhuis SKB , Hoekema A , et al. Long‐term oral appliance therapy in obstructive sleep apnea syndrome: a controlled study on temporomandibular side effects. Clin Oral Investig. 2012;16:689–697. 10.1007/s00784-011-0555-6 21538074

[31] Walker‐Engström M‐L , Tegelberg A , Wilhelmsson B , Ringqvist I . 4‐year follow‐up of treatment with dental appliance or uvulopalatopharyngoplasty in patients with obstructive sleep apnea: a randomized study. Chest. 2002;121:739–746. 11888954 10.1378/chest.121.3.739

[32] Giannasi LC , Almeida FR , Magini M , et al. Systematic assessment of the impact of oral appliance therapy on the temporomandibular joint during treatment of obstructive sleep apnea: long‐term evaluation. Sleep Breath. 2009;13:375–381. 19437057 10.1007/s11325-009-0257-3

[33] de Almeida FR , Lowe AA , Tsuiki S , et al. Long‐term compliance and side effects of oral appliances used for the treatment of snoring and obstructive sleep apnea syndrome. J Clin Sleep Med. 2005;1:143–152. 17561628

[34] Alessandri‐Bonetti A , Bortolotti F , Moreno‐Hay I , et al. Effects of mandibular advancement device for obstructive sleep apnea on temporomandibular disorders: a systematic review and meta‐analysis. Sleep Med Rev. 2019;48:101211–101211. 31605905 10.1016/j.smrv.2019.101211

[35] Bartolucci ML , Bortolotti F , Martina S , Corazza G , Michelotti A , Alessandri‐Bonetti G . Dental and skeletal long‐term side effects of mandibular advancement devices in obstructive sleep apnea patients: a systematic review with meta‐regression analysis. Eur J Orthod. 2019;41:89–100. 29901715 10.1093/ejo/cjy036

[36] de Almeida FR , Bittencourt LR , de Almeida CIR , Tsuiki S , Lowe AA , Tufik S . Effects of mandibular posture on obstructive sleep apnea severity and the temporomandibular joint in patients fitted with an oral appliance. Sleep. 2002;25:507–513. 12150316

[37] Ruf S , Pancherz H . Temporomandibular joint remodeling in adolescents and young adults during Herbst treatment: A prospective longitudinal magnetic resonance imaging and cephalometric radiographic investigation. Am J Orthod Dentofac Orthop. 1999;115:607–618. 10.1016/s0889-5406(99)70285-4 10358242

[38] Ruf S , Pancherz H . Long‐term TMJ effects of Herbst treatment: a clinical and MRI study. Am J Orthod Dentofacial Orthop. 1998;114:475–483. 9810042 10.1016/s0889-5406(98)70166-0

[39] Adhikari H , Prakash U , Kapoor A , Srivastava A . “Electromyographic pattern of masticatory muscles in altered dentition” Part II. J Conserv Dent. 2011;14:120. 21814350 10.4103/0972-0707.82607 PMC3146101

[40] Alessandri‐Bonetti G , Bortolotti F , Bartolucci ML , Marini I , D’Antò V , Michelotti A . The effects of mandibular advancement device on pressure pain threshold of masticatory muscles: a prospective controlled cohort study. J Oral Facial Pain Headache. 2016;30:234–240. 27472526 10.11607/ofph.1500

[41] Masse J‐F , Bellerive A , Sériès F , St‐Pierre L . Prospective assessment of maximum protrusion in patients wearing a mandibular advancement device. JDSM. 2018;05:11–16.

[42] Kaimal S , Ahmad M , Kang W , Nixdorf D , Schiffman EL . Diagnostic accuracy of panoramic radiography and MRI for detecting signs of TMJ degenerative joint disease. Gen Dent. 2018;66:34–40. PMC9488601 29964246

